# Mitral annular calcification predicts immediate results of percutaneous transvenous mitral commissurotomy

**DOI:** 10.1186/1476-7120-9-29

**Published:** 2011-10-28

**Authors:** Mojtaba Salarifar, Mehrnaz Rezvanfard, Hakimeh Sadeghian, Azam Safir-mardanloo, Nahid Shafii

**Affiliations:** 1 Interventional Department, Tehran Heart Center, Tehran University of Medical Sciences, Tehran, Iran; 2 Research Department, Tehran Heart Center, Tehran University of Medical Sciences, Tehran, Iran; 3 Echocardiography Department, Tehran Heart Center, Tehran University of Medical Sciences, Tehran, Iran

**Keywords:** Percutaneous transvenous mitral commissurotomy (PTMC), Mitral annular calcification (MAC), PTMC result, Mitral valve morphology

## Abstract

**Background:**

Many previous studies have evaluated the impact of mitral valve (MV) deformity scores on the percutaneous transvenous mitral commissurotomy (PTMC) outcome in patients with mitral stenosis; however, the relationship between mitral annulus calcification (MAC) and the PTMC result has not yet been established. The current study aimed to investigate whether MAC could independently influence the immediate result of PTMC.

**Methods:**

Of all patients undergoing PTMC in our institution between April 2005 and November 2009, we included 87 patients (28.7%male, mean ± SD age = 42.8 ± 12.6 years) with rheumatic mitral stenosis who had additional data on the echocardiographic evaluation of MAC along with MV leaflets morphology. Echocardiographic assessments were repeated up to six weeks after PTMC to evaluate the immediate PTMC outcome. The frequency of the optimal PTMC result (secondary MV area > = 1.5 cm^2 ^with > = 25% increase and without final mitral regurgitation grade > 2) was compared between two groups of patients with MAC (n = 17) and those without MAC (n = 70).

**Results:**

The optimal result was obtained in 55 (63.2%) patients, whereas the result was suboptimal in 32 (36.8%) patients due to insufficient MV area increase in 31(96.9%) subjects and post-procedure mitral regurgitation grade > 2 in 1(3.1%). The rate of optimal PTMC results was less in patients with MAC in comparison to those without MAC (29.4% vs.71.4%). After adjustments for possible confounders such as age and leaflets morphological subcomponents (thickening, mobility, calcification, and subvalvular thickening), MAC remained a significant negative predictor of a suboptimal PTMC result (odds ratio = 0.154; 95%CI = 0.038-0.626, p value = 0.009) together with leaflet thickening (odds ratio = 0.214; 95%CI = 0.060-0.770, p value = 0.018).

**Conclusions:**

MAC appeared to independently influence the immediate result of PTMC; therefore, mitral annulus evaluation may be considered in the echocardiographic assessment of the mitral apparatus prior to PTMC.

## Introduction

percutaneous transvenous mitral commissurotomy (PTMC) has become established as a procedure of choice for the treatment of mitral stenosis (MS) [1-3] and it confers equivalent results to open and closed surgical valvotomy in patients whose valves are anatomically suitable [4-6]. Appropriate patient selection, however, is of paramount importance for a successful PTMC.

In the selection of patients for PTMC, the echocardiographic assessment of the mitral valve morphology plays a crucial role [7-10] and it is now performed routinely in most centers. The Wilkins score is one of the most widely used echocardiographic scoring systems [7,9,11,12] in that it provides a semi-quantitative assessment of mitral leaflets thickening, mobility, calcification, and the extent of the subvalvular apparatus disease. A favorable Wilkins score (< 8 points) is highly predictive of an optimal outcome after PTMC [3,7,9]. Nevertheless, there are studies which have questioned the precision of this score as a predictor of the outcome and have suggested the need for more refined and comprehensive echocardiographic assessments [1,11,13,14]. Likewise, the Wilkins scoring system does not examine mitral annular calcification (MAC), which is characterized by calcium and lipid deposition within the annular fibrosa of the mitral valve [15,16] and might independently influence the PTMC result as it appears to be a different feature from leaflets or commissural calcification in terms of the incidence rate, underlying predisposing factors, pathophysiology, and associative cardiovascular disorders or systemic comorbidities [15-21].

To our knowledge, there is not enough studies evaluating the impact of MAC on the PTMC immediate result. The current study aimed to investigate if pre-procedure echocardiographic evaluation of MAC could help the clinician to predict the immediate result of PTMC.

## Methods

### Study Population

From April 2005 to November 2009, PTMC was attempted in 153 consecutive patients with the diagnosis of rheumatic MS at Tehran heart center according to previously established criteria [22]. Patients population were those referred from inside cardiology clinics or directly from outside physicians. Pre-procedure conventional echocardiography conducted in all patients to investigate the MV morphology in our echocardiographic units; however, we just included 89 consecutive patients who underwent echocardiographic evaluation in one of our units equipped by Vivid-7 (Vingmed GE) echocardiography apparatus and had additional data on the echocardiographic evaluation of the mitral annulus. After excluding two cases (because of the previous history of open or closed mitral commissurotomy) retrospective analysis conducted on the data of the final 87 patients [mean ± SD age = 42.8 ± 12.6 years and 25 (28.7%) male]. PTMC was contraindicated in the presence of left atrial thrombus, significant coexistent valve lesions, bilateral commissural calcification, and mitral regurgitation (MR) greater than grade 2+ and unfavorable MV morphology with Wilkins total score > 12 as estimated by echocardiography. The relation between the echocardiographic score of the valve morphology and PTMC immediate result was assessed by defining the optimal result as mitral valve area (MVA) ≥ 1.5 cm^2 ^or more and increase in the MVA of at least 25% without post-procedure MR grade > 2. This definition was employed on account of the fact that it is one of the most commonly employed criteria in the existing literature [3,23]. The study protocol was approved by the Ethics Committee Review Board of the hospital.

### Echocardiographic Evaluation

**Figure 1:**
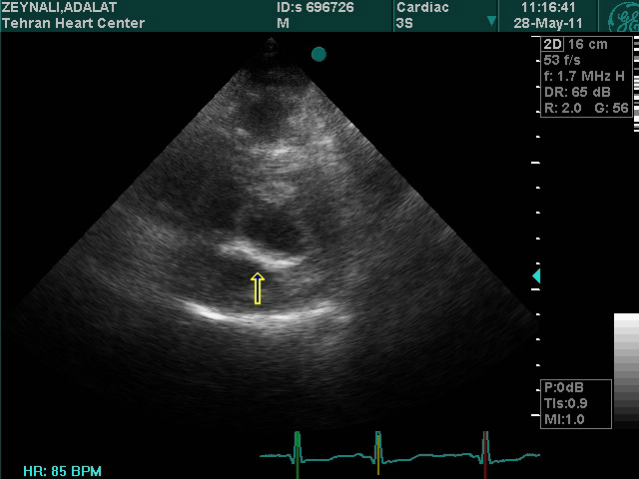
**Parasternal short-axis view of mitral annulus calcification (MAC) by transthoracic echocardiography**.

**Figure 2:**
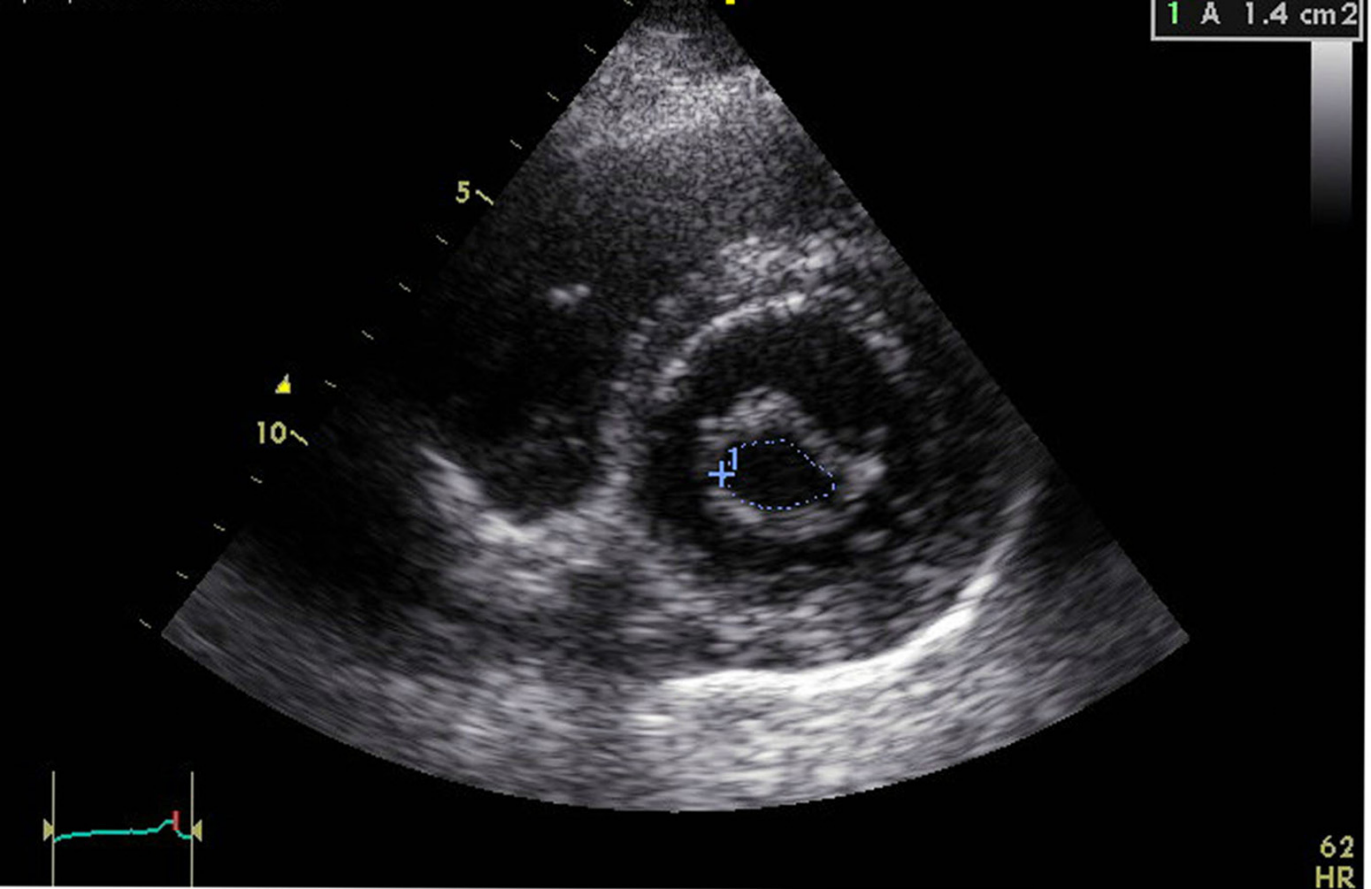
**Calculation of mitral valve area(MVA) via planimetry method from parasternal short-axis view in one patient without mitral annulus calcification(MAC) prior PTMC**.

**Figure 3:**
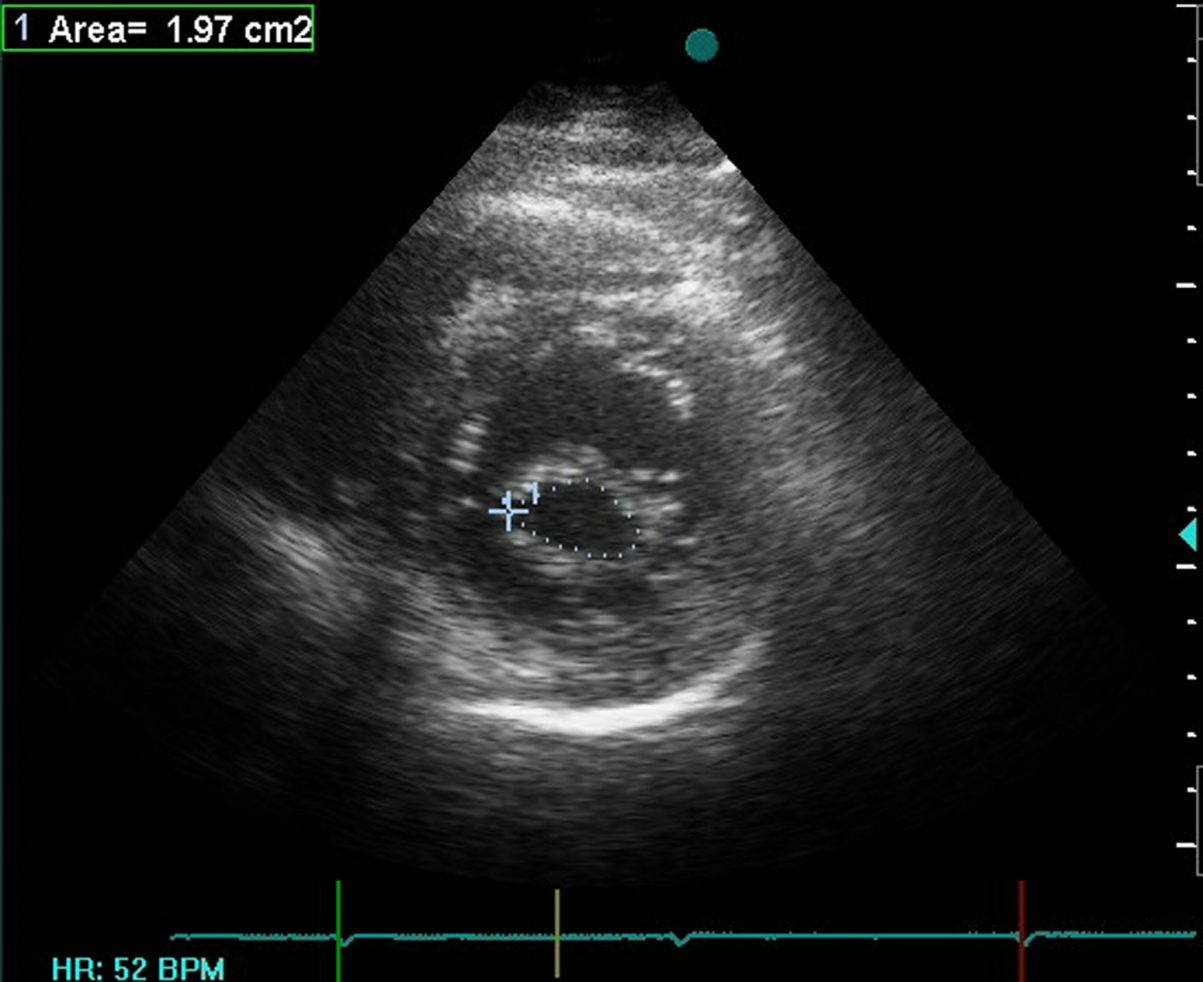
**Calculation of mitral valve area(MVA) via planimetry method from parasternal short-axis view in one patient without mitral annulus calcification(MAC) after PTMC**.

**Figure 4:**
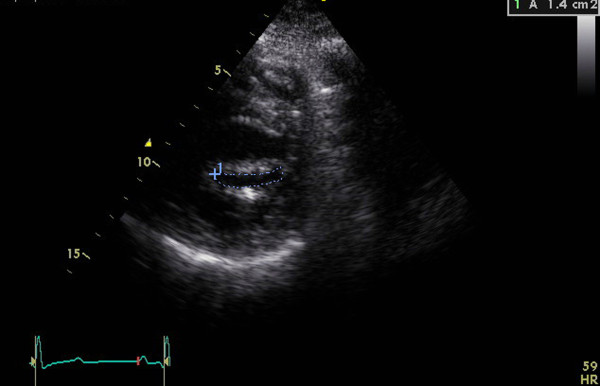
**Calculation of mitral valve area(MVA) via planimetry method from parasternal short-axis view in one patient with mitral annulus calcification(MAC) prior PTMC**.

**Figure 5:**
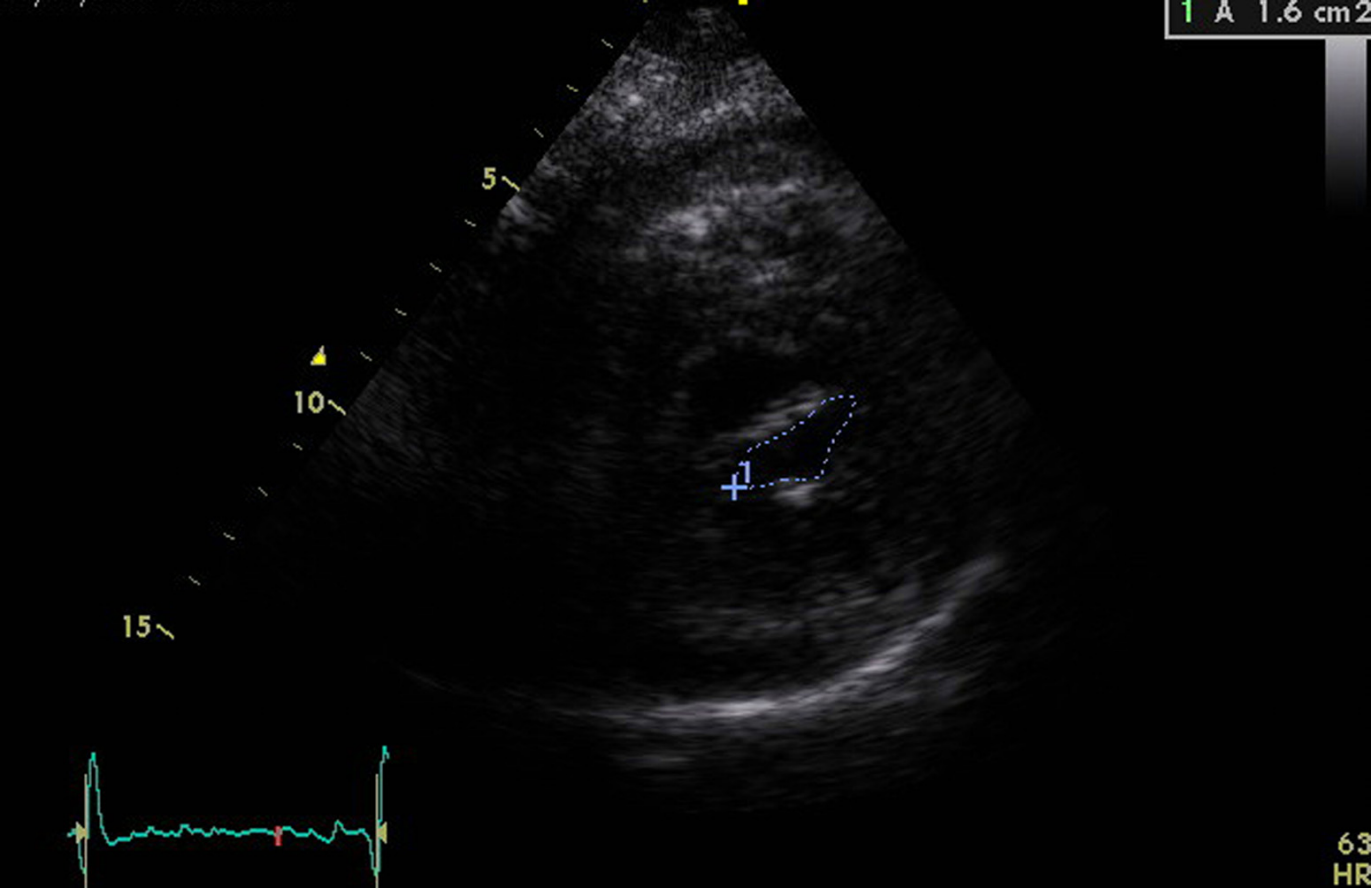
**Calculation of mitral valve area(MVA) via planimetry method from parasternal short-axis view in one patient with mitral annulus calcification(MAC) after PTMC**.

Echocardiographic assessments were conducted in all the patients during the week leading up to the procedure using a combination of transthoracic two-dimensional (2D), pulsed and continuous-wave Doppler with color-flow imaging (Vingmed GE, Horten, Norway, 3.5-MHz transducer), and transesophageal echocardiography (Vivid-7, Vingmed GE, Horten, Norway, 7-MHz transducer). From the echocardiographic study, the MVA was calculated via planimetry [See additional file 1 and additional file 2] and MR severity was scored 0 as no or trivial, 1 as mild, 2 as moderate, 3 as moderate to severe, and 4 as severe [24]. The morphological features of the mitral valve (MV) were scored individually in terms of (a) leaflet thickening, (b) leaflet mobility, (c) leaflet calcification, and (d) subvalvular thickening according to the Wilkins scoring system [7]. Each subcomponent had a possible score of 0 to 4 corresponding to zero to severe abnormality. The presence of calcification of the mitral annulus and commissures determined the use of 2D-echocardiograms in the parasternal short-axis view. MAC was defined as a dense, highly reflected area at the base of the posterior mitral leaflet [15] (Figure 1), while anterolateral and/or posteromedial commissure calcification was identified by bright and confluent echo which was brighter than the adjacent aortic root [25]. A secondary transthoracic echocardiography evaluation was performed for all the patients between twenty-four hours and six weeks after the procedure in order to evaluate the hemodynamic change and immediate PTMC outcome [See additional file 3 and additional file 4]. Figure 2 and 3 show the MVA change in a patients without MAC prior and following the PTMC while Figure 4 and 5 show the MVA change in a patient with MAC.

### Cardiac Catheterization and Percutaneous Transvenous Mitral Commissurotomy

A self-positioning single balloon (Inoue balloon) was applied for commissurotomy in all the patients. The upper limit of the balloon diameter was chosen according to the patient's height. Inflation was commenced at less than the predetermined upper-limit diameter. If the hemodynamic results were suboptimal, the procedure was repeated by gradually increasing the balloon diameter until the reduction in the transmitral gradient was satisfactory. If optimal hemodynamic results were not achieved at the maximum diameter of the balloon, additional inflation was not attempted as a general rule.

### Statistical Methods

The data are presented as mean ± SD (standard deviation) for the numerical variables and are summarized by absolute frequencies and percentages for the categorical variables. A univariate analysis of the baseline demographic data and echocardiographic measurements was performed within the individual groups applying the Student *t*-test, Mann-Whitney *U *test, and Fisher exact test, as appropriate.

Multiple logistic regression analysis was employed to appraise the association between MAC and the immediate PTMC result (binary variables were defined as optimal and suboptimal) when simultaneously controlled for the known parameters which might affect the PTMC result. Indeed, the presence or absence of MAC along with age (years) and MV morphological subcomponents (thickening, calcification, mobility, and subvalvular thickening) were all entered into the multivariable model. Finally, the associations between the independent predictors of the optimal result were expressed as odds ratios (OR) with 95% CIs. Model discrimination was measured using the statistic, which is equal to the area under the ROC (Receiver Operating Characteristic) curve. Model calibration was estimated using the Hosmer-Lemeshow goodness-of-fit statistic (higher p values imply that the model fits the observed data better). The Pearson coefficient was utilized to investigate the association between the various subcomponents of the Wilkins scoring system. For the statistical analysis, the statistical package SAS version 9.1 for Windows (SAS Institute Inc., Cary, NC, USA) was used. All the p values were two-tailed, with statistical significance defined by a p value ≤ 0.05. Intra- and inter-observer variability was analyzed in 10 randomly selected subjects and expressed as the mean percentage error (difference/mean) for the numerical variables and the Cohen Kappa coefficient for the categorical variables.

## Results

The PTMC procedure was successfully completed in all the patients, and there were no deaths or complications associated with the procedure. The optimal result was obtained in 55 (63.2%) patients, whereas the result was suboptimal in 32 (36.8%) patients [due to insufficient MV area increase in 31(96.9%) subjects and post-procedure MR grade > 2 in 1 (3.1%0 case)]. The mean MVA increased significantly from 1.0 ± 0.2 cm^2 ^(range = 0.6-1.4) to 1.8 ± 0.2 cm^2 ^(range = 1.5-2.6) (p value < 0.001) in patients with the optimal result. After the procedure, echocardiographic development in MR grade developed in one patient to moderate to severe level. As the patient was hemodynamically stable, she was followed up on pharmacological treatment with diuretic, ACE inhibitors and digoxin without any need to urgent surgical mitral valve replacement therapy. Hemodynamic evaluation showed a significant decline in the mean of the MV mean gradient (MVMG) (from 11.8 ± 5.7 to 5.0 ± 2.0 mmHg, p value < 0.001), MV peak gradient (MVPG) (from 19.9 ± 8.0 to 9.3 ± 3.2 mmHg, p value < 0.001), and pulmonary artery systolic pressure (PAPs) (from 44.7 ± 11.5 to 35.0 ± 9.3 mmHg, p value < 0.001) in patients with the optimal result.

**Figure 6:**
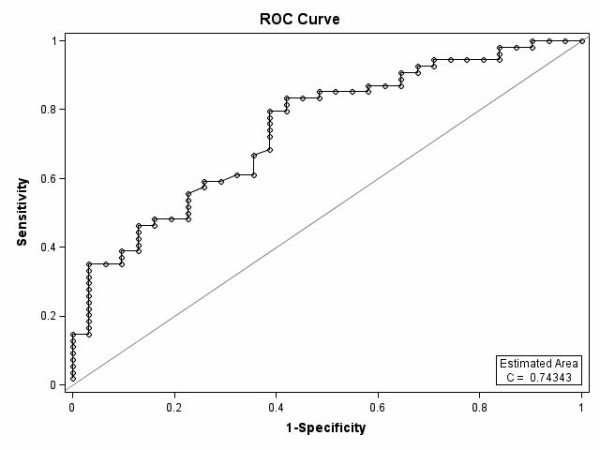
**The ROC curve derived from factors affecting the optimal result following PTMC**.

According to Table 1, 17 (19.5%) patients exhibited echocardiographic evidence of MAC, while the mitral annulus was normal in 70 (80.5%) patients. The total Wilkins score ranged from 7 to 12 in patients who had evidence of calcification in the mitral annulus. The rate of the optimal PTMC result was significantly lower in patients with MAC than in those with a normal mitral annulus [5(29.4%) vs. 50(71.4%), p value < 001]. The baseline demographic and hemodynamic data were not different between these two groups; however, the MV morphological evaluation indicated a higher frequency of leaflets abnormalities in patients with MAC. In particular, the Wilkins total score and two of its subcomponents (calcification and subvalvular thickness) were significantly and the thickness score tended to be significantly higher in patients with MAC than the corresponding scores in patients without MAC. After adjustments for age and the MV deformities subcomponents (thickness, calcification, mobility, and subvalvular thickness), which constitute the previously known parameters that might influence the PTMC outcome, patients with MAC were still at an increased risk of a suboptimal PTMC result (OR = 0.154, 95%CI = 0.038-0.626, p value < 0.01). MV leaflet thickness was also identified as an independent negative predictor of the PTMC result (OR = 0.214, 95%CI = 0.060-0.770, p value < 0.05). The good fit of the model was shown by the absence of a significant difference between the predicted (using the model) and observed results (Hosmer-Lemeshow goodness-of-fit test, p value = 0.33). The area under the ROC curve reached 0.74343 (Figure 6).

**Table 1:** Echocardiographic assessment of mitral valve apparatus morphology in patients undergoing percutaneous transvenous mitral commissurotomy (PTMC)

	Total (n = 87)	Annulus calcification		
	
		Yes (n = 17)	No (n = 70)	P value
**Age**	43.3 ± 12.3	46.4 ± 12.1	42.5 ± 12.4	0.246
**Male gender**	25(28.7)	7(41.2)	18(25.7)	0.206
**AF rhythm**	23(27.7)	5(31.3)	18(26.9)	0.725
**Optimal PTMC result**	55(63.2)	5(29.4)	50(71.4)	0.002
**Unilateral commissure calcification**	13(14.9)	6(10.9)	7(21.9)	0.167
**Mitral leaflets morphological score**				
Total	8.1 ± 1.2	8.8 ± 1.2	8.0 ± 1.1	0.013
Thickness	2.1 ± 0.4	2.3 ± 0.5	2.1 ± 0.4	0.089
Calcification	1.5 ± 0.6	1.8 ± 0.7	1.4 ± 0.6	0.049
Subvalvular thickening	2.4 ± 0.6	2.4 ± 0.5	2.4 ± 0.6	0.685
Mobility	2.1 ± 0.3	2.2 ± 0.3	2.1 ± 0.3	0.024
**Pre-PTMC echocardiographic measurements**				
Pre-PTMC MVA	1.0 ± 0.2	0.9 ± 0.2	1.0 ± 0.2	0.533
Mitral regurgitation (MR) grade	0.8 ± 0.6	0.8 ± 0.5	0.8 ± 0.6	0.803
Left ventricle ejection fraction (%)	54.5 ± 4.5	54.8 ± 6.7	54.5 ± 3.9	0.787
Mitral valve peak gradient (MVPG)(mmHg)	19.9 ± 7.7	22.2 ± 7.9	19.4 ± 7.6	0.169
Mitral valve peak gradient (MVMG)(mmHg)	12.3 ± 6.0	13.8 ± 5.8	11.9 ± 6.0	0.253
Systolic left ventricle dimension (sLVD)(mm)	31.9 ± 6.5	31.8 ± 6.9	31.9 ± 6.4	0.962
Diastolic left ventricle dimension (dLVD)(mm)	45.2 ± 6.1	47.4 ± 1.1	45.8 ± 4.6	0.121
Left atrium size (LAs) (mm)	45.4 ± 7.3	43.5 ± 7.6	45.8 ± 7.3	0.256
Pulmonary artery systolic pressure (PAPs) (mmHg)	46.0 ± 12.5	49.8 ± 9.3	45.0 ± 13.1	0.174
**Post-PTMC echocardiographic measurements**				
Post-PTMC MVA (cm^2^)	1.6 ± 0.4***	1.4 ± 0.4	1.6 ± 0.4	0.038
Mitral regurgitation (MR) grade	1.0 ± 0.7	0.8 ± 0.5	1.0 ± 0.7	0.350
Left ventricle ejection fraction (%)	54.8 ± 7.6	55.6 ± 6.8	54.6 ± 7.8	0.635
Mitral valve peak gradient (MVPG)(mmHg)	11.0 ± 5.2***	10.9 ± 4.6	11.1 ± 5.4	0.903
Mitral valve peak gradient (MVPG)(mmHg)	6.0 ± 3.1***	6.2 ± 2.2	6.0 ± 3.3	0.805
Systolic left ventricle dimension (sLVD)(mm)	30.0 ± 6.4	31.5 ± 6.9	29.6 ± 6.3	0.292
Diastolic left ventricle dimension (dLVD)(mm)	45.6 ± 5.7	46.5 ± 5.3	45.4 ± 5.8	0.509
Left atrium size (LAs) (mm)	45.1 ± 9.2	45.5 ± 13.8	45.0 ± 7.9	0.846
Pulmonary artery systolic pressure (PAPs) (mmHg)	38.9 ± 13.0**	34.9 ± 9.2	40.1 ± 13.8	0.228

MVA: mitral valve area,**: p value < 0.01 and ***: p value < 0.001 in comparison with corresponding variable assessed prior to the PTMC

The impact of each MV morphological sub-score on the total score of Wilkins was weighed by evaluating the Pearson association between these scores and the total score. As it can be seen from Table 2, leaflet calcification contributes the most to the total score (r = 0.713, p value < 0.01).

**Table 2:** The Pearson correlation coefficients between various morphological sub-scores of mitral leaflets, as assessed by echocardiography

	Total score	Thickness	Calcification	Mobility	Subvalvular thickening
**Total score**	1.0				
**Thickness**	.608**	1.0			
**Calcification**	.713**	.267*	1.0		
**Mobility**	.315**	*-.028*	*.145*	1.0	
**Subvalvular thickness**	.615**	*.205*	*.067*	*-.021*	1.0

* P value ≤ 0.05, **P value ≤ 0.01

### Inter- and intraobserver variability

The echocardiographic parameters measured by the same observer were replicable for the MV morphological components (Kappa ranging from 0.58 to 0.68, approximate significance < 0.01) and MR severity (Kappa = 0.72, approximate significance < 0.01). Intraobserver variability was 7.5% for the Wilkins total score, 11.9% for MV area. The measurements taken by two echocardiographers were reproducible for the MV morphological subcomponent scores (Kappa ranging from 0.48 to 0.62, approximate significance < 0.01) and MR severity (Kappa = 0.67, approximate significance < 0.01). The interobserver variability was 11.1% for measuring the Wilkins total score, 6.4% for MV area.

## Discussion

The current study identified MAC and MV leaflet thickness as two negative predictors of the immediate PTMC result and thus suggests that a pre-procedure echocardiographic evaluation of the morphology of the MV leaflets along with mitral annulus could help have a better prediction of the PTMC outcome.

Valvular and coronary artery calcification share many similar risk factors and pathophysiological characteristics [26,27]. Aortic valve calcification(AVC) and MAC are also proposed as independent predictive factors of cardiovascular disease(CVD) events and all-cause mortality [28,29]. Calcification of the annulus fibrosus of the MV is a frequent finding particularly in older patients [16,30], but the pathophysiology of MAC is still a subject of controversy [15,18,19]. The most common hypothesis is the progressive degeneration of the annulus through life [16]; however, MAC can be also seen in patients without any primary valve tissue disorders. Biochemical factors (hypertension and metabolic disease) also play a role [16,17] in a great majority of cases and there are several studies comprising a large Multi-Ethnic Study of Atherosclerosis (MESA) conducted on 6814 patients which indicated the higher rates of AVC and MAC in patients with end-stage renal disease [28,31,32]. Rheumatic infection is a common cause of annulus calcification, which usually could affect commissures and leaflets coincidentally. Extension of calcium to the annulus usually occurs over the years in these patients [33].

MAC per se may accelerate an extensive degenerative process through a vicious cycle; it may reduce leaflet mobility, leading to secondary MV insufficiency and triggering the degenerative process through increasing tensions on the valvular structures. Likewise, our findings showed that patients with MAC significantly scored more than those with a normal mitral annulus in terms of MV deformity sub-scores and in particular, leaflet mobility, leaflet calcification, and leaflet thickness. However; explanation of the mechanism of the impact of MAC on PTMC results requires further studies evaluating the role of the underlying etiology of MAC, its severity, and its extension level to the adjacent parts

In regard to the predictive role of the Wilkins echocardiographic measurements in the immediate outcome of PTMC, the existing literature abounds with controversies. Whereas some investigators have identified the echocardiographic characteristics of the MV as a significant predictor of MV area increase [7,8,23,34], others have rejected any correlation between the MV morphology as assessed by echocardiography and MV area change [35,36]. With respect to the MV morphological subcomponents, there are studies that indicate a significant association between MV area increase and valvular thickening [9], subvalvular disease [37], and leaflet calcification [11,38], while there are reports that question the predictive role of leaflet thickness [39,40], subvalvular pathology [40], valvular calcification [2] and leaflet mobility [40] on the immediate result of PTMC. Considerable mismatch between the inclusion criteria, adoption of various definitions for a successful outcome of PTMC, and variability in balloon types have all rendered comparisons difficult or even impossible.

To sum up, pre-PTMC echocardiographic evaluation of the mitral annulus in tandem with the assessment of the MV leaflets by Wilkins scoring system appear to be helpful for clinicians to make more accurate predictions about the PTMC immediate result prior to the procedure. The presence of MAC might be regarded as a parameter which could adversely influence the PTMC immediate. Designing prospective studies with the aim of quantifying the impact of MAC severity and extension on both PTMC immediate and long-term results would help the future clinicians to better select patients for PTMC.

## Limitations

Morphological abnormalities may have been underestimated through the use of transthoracic echocardiography since we evaluated three-dimensional structures in two-dimensions. Designing prospective studies for a precise assessment of individual anterior and posterior parts of the mitral apparatus using 3-D or 4-D echocardiographic images in large population samples may provide a more accurate formula for a more accurate prediction of the PTMC result based on morphological abnormalities. Finally, it should be noted that these findings on the role of MAC in the PTMC outcome could not be generalized to the late PTMC result. Future studies based on long-term echocardiographic follow-up data would be helpful to determine whether the effect of MAC is only temporary or lasts long.

## Competing interests

The authors declare that they have no competing interests.

## Authors' contributions

HS conceived of the study and designed the study. MS participated in its design and coordination. AS participated in echocardiographic assessment of the patients. NS participated in preparing patients' data sheets and MR performed the statistical analysis and drafted the manuscript. All authors read and approved the final manuscript.

## Supplementary Material

Additional file 1 **Pre-PTMC echocardiographic assessments in a patient without MAC**. The file contains a short clip and three echocardiographic images which show MVA measurement by planimetry and PHT methods in a patient without MAC prior the PTMC. Click here for file

Additional file 2 **Pre-PTMC echocardiographic assessments in a patient with MAC**. The file contains a short clip and four echocardiographic images which show MVA measurement by planimetry and PHT methods in a patient with MAC prior the PTMC. Click here for file

Additional file 3 **Post-PTMC echocardiographic assessments in a patient without MAC**. The file contains a short clip and three echocardiographic images which show MVA measurement by planimetry and PHT methods in a patient without MAC after the PTMC Click here for file

Additional file 4 **Post-PTMC echocardiographic assessments in a patient with MAC**. The file contains a short clip and four echocardiographic images which show MVA measurement by planimetry and PHT methods in a patient with MAC after the PTMC. Click here for file
